# President’s Address

**Published:** 1905-06-15

**Authors:** A. L. Le Gro


### PRESIDENT'S ADDRESS.

BY A. L. LEGRO, D.D.S.

Delivered before the Southwestern Michigan Dental Society, April 11, 1905.

*Gentlemen of the Southwestern Michigan Dental Society:*

In addressing this society to-day, I do so with a sense of deep appreciation, I assure you, of the high honor you have bestowed upon me by electing me to the Presidency of the Southwestern Michigan Dental Society. I can not eulogize this organization too much at this time, but I realize its great power for good and its progress in this part of the State of Michigan. It is certainly gratifying to know that the men who take an active interest in the welfare of this organization, by attending the meetings and by consenting to appear in the programs, are the best and most representative of their respective communities. The man who hibernates in his office, as it were, and depends on his own limited environment to better his condition professionally, or who sees nothing worth while in the efforts of his brothers, and knows, without investigating, that his way is best, has confined his life to a narrow groove which becomes narrower and narrower until lost in darkness. Progress is essential to vitality and we should rejoice that with every succeeding year we are standing higher as a profession, because of the work done by our highly-developed dental societies.

The success of a dental society does not depend wholly upon its officers, but upon the members as well, and I want to impress with all the strength that in me lies upon every dentist in Southwestern Michigan, that if he expects to get the good that there is in the practice of dentistry, all that is new and progressive, it is up to him to encourage by his individual efforts all dental society work he can. Nearly every dentist has some good ideas that the rest of us do not possess; if he is a good professional brother, and alive to his duty, he will contribute in a small way, it may be, but in the aggregate to the general good of all.

Contrary to our regular custom, this society postponed the last meeting which was to have been held at Benton Harbor in September. The World's Dental Congress was in session at St. Louis and a great many members of this society desired to attend that meeting. Owing to this meeting, clinicians and other participants in dental society matters were hard to obtain. There seems to be a growing feeling among members of this society that one meeting in the spring of the year is enough, and that a second meeting in the same year seems unnecessery. I urge upon you a careful consideration of this particular point that you may definitely decide the advisability of such a change. I would suggest that this society arrange joint meetings with the Northern Indiana and Central Michigan Dental Societies should it conclude to maintain two meetings each year. I especially recommend that we establish some kind of closer relationship with the Michigan Dental Association on such terms as the parent society may hereafter arrange. It has occurred to me that the societies of this State are entirely too distinct and isolated to bring about the most harmonious results, that would surely come if we were enlisted in one common cause. In unity there is strength, and if we are to expect the recognition from other professions of highly-educated and scientific bodies we must be well organized to command it. The men who stand high in all professions or in any community are looked upon as a part of that community's most valuable assets, It should be so in dental societies. The fact that these societies are composed of well-trained men with a high and sensitive standard of professional honor and duty is of incalculable worth in the better organization of the profession. I trust that our proceedings may be dignified and of sufficient interest and value to merit the large attendance, which I most cordially invite to a free participation in our program.

